# Numerical Modelling of Concrete-to-UHPC Bond Strength

**DOI:** 10.3390/ma13061379

**Published:** 2020-03-18

**Authors:** Alireza Valikhani, Azadeh Jaberi Jahromi, Islam M. Mantawy, Atorod Azizinamini

**Affiliations:** 1 Department of Civil and Environmental Engineering Department, Florida International University, Miami, FL 33172, USA; imantawy@fiu.edu (I.M.M.); aazizina@fiu.edu (A.A.); 2 Washington State Department of Transportation, Olympia, WA 98505, USA; Jabera@wsdot.wa.gov

**Keywords:** UHPC, interface, bond strength, numerical analysis

## Abstract

Ultra-High Performance Concrete (UHPC) has been a material of interest for retrofitting reinforced concrete elements because of its pioneer mechanical and material properties. Numerous experimental studies for retrofitting concrete structures have shown an improvement in durability performance and structural behaviour. However, conservative and sometimes erroneous estimates for bond strength are used for numerically calculating the strength of the composite members. In addition, different roughening methods have been used to improve the bond mechanism; however, there is a lack of numerical simulation for the force transfer mechanism between the concrete substrate and UHPC as a repair material. This paper presents an experimental and numerical programme designed to characterize the interfacial properties of concrete substrate and its effect on the bond strength between the two materials. The experimental programme evaluates the bond strength between the concrete substrates and UHPC with two different surface preparations while using bi-surface test and additional material tests, including cylinder and cube tests for compression property, direct tension test, and flexural test to complement UHPC tensile properties. Non-linear finite element analysis was conducted, which uses a numerical zero thickness volume model to define the interface bond instead of a traditional fixed contact model. The numerical results from the zero thickness volume model show good agreement with the experimental results with a reduction in error by 181% and 24% for smooth and rough interface surfaces if compared to the results from the model with a fixed contact.

## 1. Introduction

Ultra-high performance concrete (UHPC) has been in the interest of research with different range of applications for bridge construction, repair and rehabilitation, overlays, building, petroleum industry, hydraulic structures, and architectural components [1,2]. UHPC is developed by the inspiration of three concrete technologies: self-compacting concrete (SCC), high-performance concrete (HPC), and fibre reinforced concrete (FRC) with a high compressive strength that is higher than 126 MPa (18 ksi) and post-cracking tensile strength of more than 5 MPa (0.7 ksi) [1,3,4,5,6,7]. UHPC with a different mix design model is a combination of portland cement, sand, quartz powder, silica fume, superplasticizer, water, and steel fibres [4,8,9,10,11]. In the UHPC mixture, a higher portion of cement is used compared to the HPC and normal strength concrete (NSC) [12,13] and the key factor of UHPC production is the enhancement of material density, mechanical homogeneity, and particle packing by improving macro and micro properties [12,13,14,15,16,17,18,19,20], which help the durability of UHPC against chloride diffusion. The water-to-cement ratio (W/C) of UHPC is typically less than 0.25 due to the replacement of the portion of un-hydrated cement with blast furnace slag, fly ash, or crushed quartz [21,22,23,24,25]. Superplasticizer is added to the mixture to improve the workability of UHPC, which is low due to the low W/C ratio [15,16,26,27,28,29]. Adding silica fume increases the workability of the mixture and fills the voids between aggregate particles, therefore increasing the compressive strength [15,30,31,32]. Steel fibres are added to change the brittle behaviour of the mixture to a ductile behaviour. The most common size of steel fibres is 13 mm (0.5 in.) in length and 0.20 mm (0.008 in.) in diameter with a recommended ratio of 2% by volume [3,7,18,28,33]. The outstanding characteristics of UHPC enable it as a practical option for repairing and retrofitting damaged structural elements [2,34] connecting the precast elements [35,36] or as an overlay for bridge decks [37,38]. In all of these applications, sufficient interfacial bond strength between normal concrete substrates and UHPC is required to ensure the resulting section is sufficiently composite. Although the bond strength between normal concrete and UHPC has been experimentally investigated by many researchers while using bi-surface, slant shear, push off, and pull-off tests [39,40,41,42,43,44], there is a gap knowledge that is related to the numerical modelling of the interfacial bond strength for repair design and evaluation. Harris et al., [45] and Azizinamini et al., [46] presented an approach to solve this challenge using simple fix contact surface or tie model between two layers. Such an approach might cause the overestimation of bond strength and global structural performance. This paper presents a realistic approach to evaluate the interfacial bond strength between UHPC and NSC that is numerically based on experimental data using ATENA finite element (FE) software (version 5, Červenka Consulting s.r.o., Czchia, Czech Republic). Mechanical properties of UHPC are investigated to achieve this goal, and the test results are used to simulate the behaviour of UHPC in ATENA software. These three-dimensional FE models can be used to simulate the response of structures that are made of UHPC or repaired and retrofitted while using UHPC and solve the challenge of interfacial bond modelling between UHPC and NSC.

## 2. Materials and Methods

The first phase of this research included the testing of ten bi-surface shear specimens to experimentally quantify the interfacial bond between UHPC as a repair material and substrates that are made of NSC with two surface different preparations for NSC substrates (as cast and sand-blasted) [39,47]. The second phase included the development of numerical models to calibrate the interfacial bond strength between UHPC and NSC. To develop numerical models for the interfacial bond between UHPC and NSC, mechanical properties of UHPC were tested and calibrated numerically, including compressive strength, tensile strength, and flexural strength. For all the experimental tests, a universal testing machine (UTM) with 2224 kN (500 kips) maximum capacity and an Mesure Test Simulte (MTS) machine with 111 kN (25 kips) maximum capacity were used to apply the loads as testing apparatus. For the numerical modelling, a commercial finite element software, ATENA, was used because of its accurate concrete material models for fibre reinforced concrete and interface model. 

### 2.1. Material and Mixing

**Figure 1:**
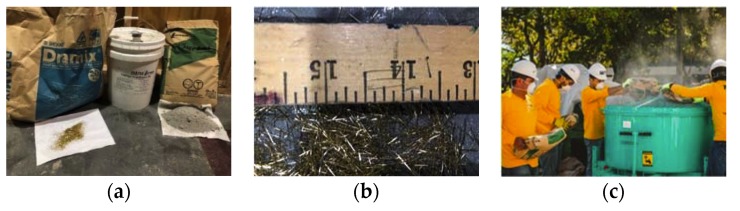
UHPC mixing process: (**a**) UHPC components; (**b**) steel fibers used in the mixture; The large pan-style mixture used for mix UHPC; and, (**c**) orbital pan-style mixer used.

This research utilized Ductal^®^ JS1000 (LafargeHolcim, Clamart, France), which is a proprietary UHPC mix that is manufactured by Lafarge. This premix includes Portland cement, fine sand, ground quartz, silica fume, and accelerator. However, the rest of the UHPC components, such as superplasticizer and steel fibres, were shipped in different packaging by the same manufacturer, as shown in Figure 1a. The superplasticizer was Chryso^®^ Fluid Premia 150 (manufacturer, city, country), and the steel fibres were straight fibres with a radius of 0.1 mm (0.004 in.) and a length of 13 mm (0.5 in.). The concentration of steel fibers in the mixture was chosen to be 2% by volume. UHPC was proportioned by weight while using a 136-kg (300-lbs) scale with an accuracy of ±0.01 kg (0.022 lbs). Water and steel fibres were individually batched using 19-L (5-gallon) buckets, and the superplasticizer was batched in a smaller plastic cup. Water and the superplasticizer were batched 20 min. before the mixing process to reduce the potential of evaporation. Table 1 lists the UHPC weight proportions.

A large orbital pan-style mixer was used to mix UHPC specimens, as shown in Figure 1c. The mixing procedure of UHPC components started by dispatching UHPC dry premix into the mixture for four minutes mixing time. The required water and half of the superplasticizer were added to the mixer for 15 min. mixing time. Subsequently, the other half of the superplasticizer was added, and, after two minutes of mixing, the dry mix turned to a concrete paste. Afterwards, the steel fibres were added to the mixture. The UHPC mixture was mixed for a further five to six minutes to have a uniform mix. Table 2 shows the mixing procedure.

### 2.2. Quality Control and Curing

**Figure 2:**
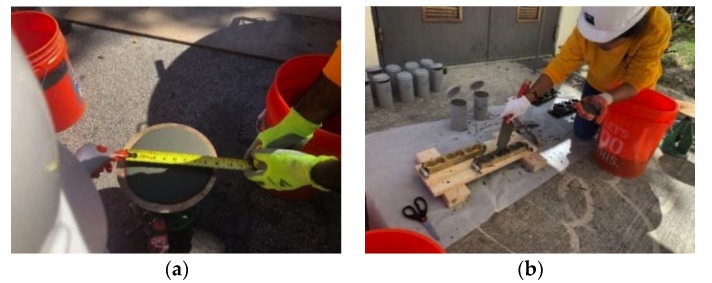
UHPC quality control: (**a**) rheological property measurement, (**b**) sampling process.

ASTM C1437 [48] and Cortes et al., [49] were used to evaluate the rheology of the fresh UHPC. In this test, the fresh UHPC was discharged in a brass cone mould, which is placed over a standard flow table with a diameter of 254 mm (10 in.). The mould was removed straightly upward to allow the fresh UHPC to flow out and settle. Subsequently, the diameter of UHPC was measured along the four perpendicular lines on the flow table, as shown in Figure 2a. The average of these diameter measurements is called static flow. The flow table was manually dripped in height for 13-mm (0.5-in.) interval 20 times, and then the average of the diameter measurement in four perpendicular directions, after 20 drops, was calculated to obtain the dynamic flow. The static flow and dynamic flow of UHPC were measured at 216 mm (8.5 in.) and 228 mm (9 in.), respectively. The rheological property of the mix was categorized as fluid based on Table 3.

The fresh UHPC was cast in moulds that were based on ASTM C1856 [50] with no need for compaction due to the high flowability and self-consolidating characteristics of UHPC [51]. The sample cylinders and cubes for compressive strength test, dog bone specimens for tensile test, beams for the flexural test, and the portion of large cubes for bond strength test were cast, as shown in Figure 2b, and as described hereafter. The sample moulds were removed after 48 h of casting and were then left in an ambient condition at a temperature of 23 ± 2 °C (74 ± 3 °F) and humidity of 50% ± 5% inside the laboratory. It should be noted that all test specimens were untreated to mimic UHPC conditions in the field [52].

## 3. Bond Strength Test

For different structural applications, such as repairing and strengthening old concrete structures or connecting full-depth deck panels using closure joints [53,54], casting UHPC next to concrete at different ages or even casting UHPC next to steel [55] highlights the challenge of bond strength between these two materials. A bi-surface shear test setup was selected to measure the bond strength for smooth and rough interface surfaces between the two materials to quantify the interfacial bond strength between UHPC and NSC [56,57].

**Figure 3:**
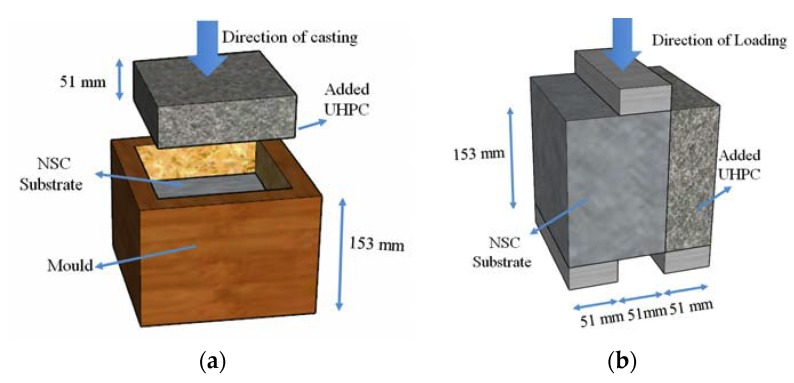
The dimension of the bi-surface shear test specimen: (**a**) casting process, (**b**) test setup (1 in. = 25.4 mm).

In this paper, ten cubical specimens of 153 mm (6 in.) sides were cast. The concrete substrate portion of the cube occupies two-third of the volume; however, UHPC occupies the other third, as shown in Figure 3. It should be noted that in the first stage of casting, NSC was cast and, in the second stage, UHPC was added like an overlay after 56 days. These specimens were divided into two groups that were based on interface surface preparation. In the first group, the concrete surface was kept as cast, hereafter referred to as “Smooth”. In the second group, the concrete surface was roughened while using sandblasting with an average surface roughness of 1.72 mm (0.068 in.), hereafter referred to as “Rough”.

Loading plate of 38 mm × 51 mm × 153 mm (1.5 in. × 2 in. × 6 in.) was used in the bi-surface shear test setup, which results in two shear planes, as shown in Figure 3. One shear plane is located at the interface between NSC and UHPC. The other shear plane is located inside the concrete substrate. The universal testing machine (UTM) was used with 935 N/s (210 lb/s) load rate, which is equal to 0.02 MPa/sec (2.92 psi/s) bond strength. The experimental bond strength is calculated using Equation (1).

(1) $$\tau =\frac{P}{2\times b\times d}$$

where *τ*: bond strength; *P*: load at failure; *b*: the width of the cube cross-section; and, *d*: the depth of the cube cross-section.

**Figure 4:**
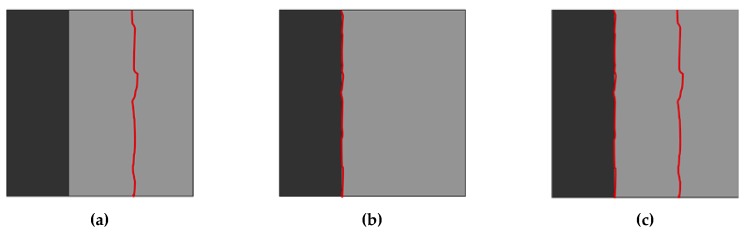
Failure modes for bi-surface shear specimens: (**a**) cohesive failure; (**b**) adhesive failure; and, (**c**) mixed failure.

**Figure 5:**
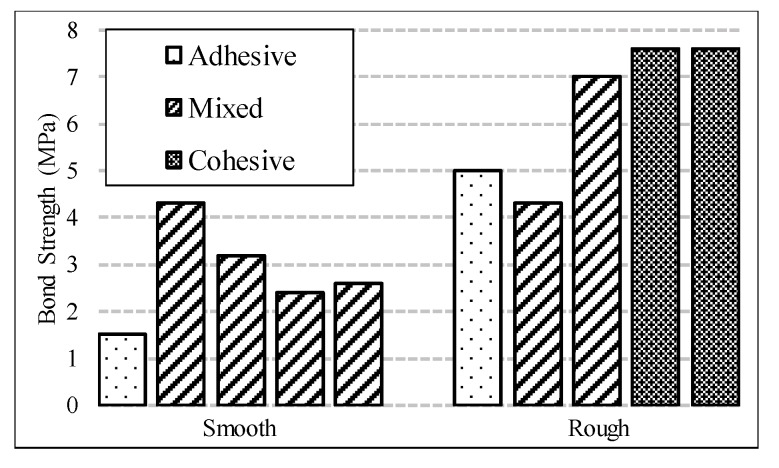
Bond strength results (1 MPa = 145 psi).

The compressive strength of NSC was measured based on the ASTM C39 [58] for six concrete cylinders of 75 mm (3 in.) in diameter and 150 mm (6 in) in height and it was measured at 43 MPa (6.2 ksi) [39]. The failure modes of each bi-surface shear specimen are divided into three categories: (1) concrete crushing, (2) debonding at the interface, and (3) concurrent failure in bond and concrete, hereafter referred to as cohesive failure, adhesive failure, and mixed failure, respectively, as shown in Figure 4. Figure 5 shows the results of the bi-surface shear test. The average bond strengths are 2.8 MPa (406 psi) for specimens with a smooth interface and 6.3 MPa (914 psi) for the specimens with a rough interface [39].

## 4. Modelling Assumptions

The ATENA software considers three-dimensional constitutive material models for simulating concrete behaviour with a combination of plasticity and fracture models [59]. Rankine tensile criterion is the base of the orthotropic smeared crack model that is used to model fracture. Menétrey and Willam (1995) [60] suggested a hardening/softening plasticity model that is used to simulate concrete crushing with a three-parameter failure surface [61] in ATENA. In this study, NSC compressive strength is used as the concrete class and all of the parameters are calculated by the software based on a fracture-plastic model. For UHPC, compression and tensile behaviours differ from NSC in tensile strength and fracture energy values and in the tensile and compression softening branch behaviour [61]. However, in ATENA software, user-defined material models with constitutive laws can be used, such as ‘‘CC3DNonLinCementitious2user’’. These constitutive laws are tensile and post-cracking softening behaviour, compression behaviour, the effect of lateral compression on tensile strength, the effect of lateral tensile strain on the compression capacity, post-cracking shear strength, and post-cracking shear stiffness [62]. Readers are advised to consult the ATENA manual for more detailed information about material constitutive laws.

Different modelling parameters are defined in this research. These parameters include tensile strength, modulus of elasticity, Poisson ratio, compressive strength, UHPC behaviour after elastic zoom, and UHPC compressive behaviour after elastic zoon. It should be noted that, in ATENA software, two parameters are defined as “characteristic length” and “localization onset”, which are defined to reduce the mesh dependency. The characteristic length is the length of strain guage that is used in the experimental test or the element size, which is used to calibrate the material [62], and the localization onset is defined as strain at maximum stress. In this research, the characteristic length is chosen as the dimension of the mesh element.

After defining the nonlinear parameters of both NSC and UHPC in the fracture-plastic model, the modelling of the UHPC-to-concrete interface for the bond test specimens can be conducted. Generally, in numerical simulation, interfaces between two layers of concrete are modelled as a fixed contact for surface or using tie models that cause the overestimation of the interfacial bond strength that might result in eliminating the sliding between the substrate and repair material. Numerically, the interface can be idealized as a zero thickness volume model that can transfer the tangential shear and normal tractions. These transfer tractions are a function of tangential displacement (*δ_t_*) and normal displacement (*δ_n_*) [63].

**Figure 6:**
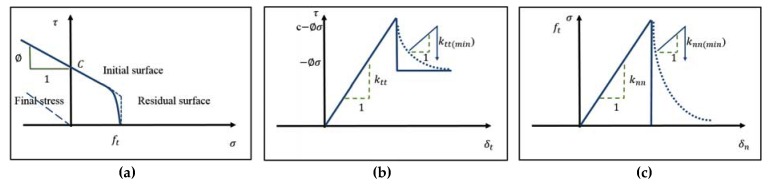
Interface modelling parameters: (**a**) failure surface for interface material based on Moher-column model; (**b**) the interface model behavior in shear; and, (**c**) the interface model behavior in tension [62].

In this study, the interface constitutive law is formulated based on the Moher-coulomb failure criterion (Figure 6), with a zero thickness volume and post-failure traction-separation laws in shear and tension [62,63].

The parameters that are shown in Figure 6 are defined, as follows: *f_t_* is the tensile strength of the bond from direct pull-off test; C is the bond cohesion measured from bond shear test; Ø is the coefficient of friction; σ is normal stress; τ is shear stress; *K_nn_* is tangent stiffness that correlates the normal displacement to the normal tractions (calibrated based on experimental results); *K_tt_* is tangent stiffness which correlates tangential displacement to the tangent tractions (calibrated based on experimental results); *K_nn(min)_* is minimum normal stiffness; and, *K_tt(min)_* is the minimum tangential stiffness.

## 5. Material Modelling Calibration

Two initial steps were conducted to simulate the interfacial bond strength between UHPC and NSC. In the first step, the fundamental characteristics of UHPC (compressive, tensile, and flexural behaviours) were experimentally tested. In the second step, the experimental results from the first step were used to calibrate and define the fracture-plastic model parameters for UHPC.

### 5.1. Compression Test

**Figure 7:**
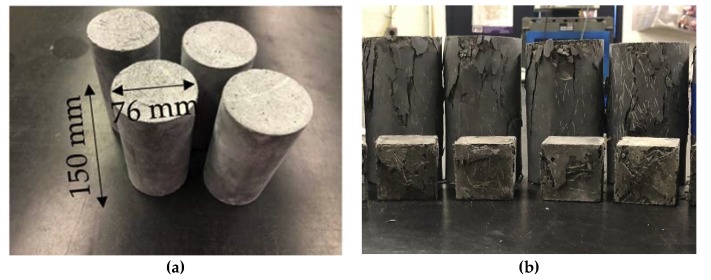
Compressive test specimens: (**a**) cylindrical specimens before the test; (**b**) cylindrical and cubical specimens after the test.

A total of nine cylindrical specimens and nine cubical specimens were cast to evaluate UHPC compressive strength and modulus of elasticity. All of the cylindrical specimens were cast in moulds of 76-mm (3 in.) in diameter and 150 mm (6 in) in height and then tested based on ASTM C39 [58]. All of the cubical specimens were cast in moulds of 51 mm (2 in.) each side and then tested based on ASTM C109 [64,65]. Both sides of cylinders’ surfaces were smooth and out of air bubbles by grinding to create a uniform pressure on the specimen’s surface during testing [66]. The length and radius of the cylinders and cube side length were measured to calculate the true stress, true strain, and density. The load rate was chosen to be 1.0 MPa/s (150 psi/s) based on federal highway administration (FHWA) recommendations [3]. Figure 7 shows the test specimens before and after testing.

**Figure 8:**
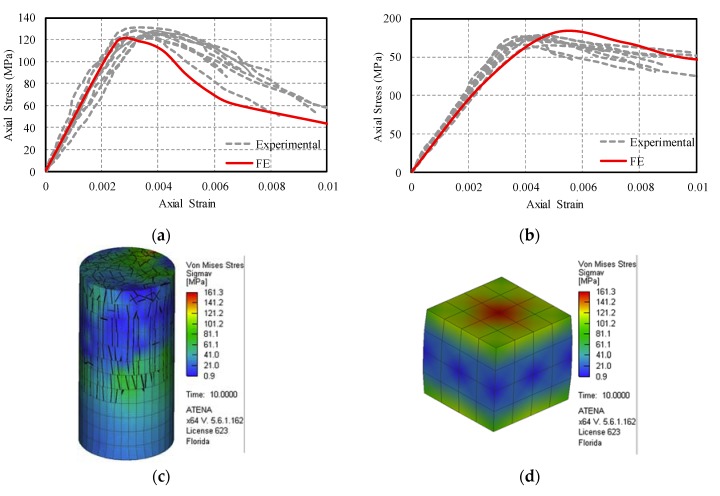
The stress-strain comparison between finite element and experimental tests: (**a**) cylindrical specimens; (**b**) cubical specimens and finite element stress distribution; (**c**) cylindrical specimen; and, (**d**) cubical specimen. (1 MPa= 145 psi).

Figure 8a,b, respectively, shows the stress-strain responses from the tested specimens for the untreated cylindrical and cubical specimens at the age of 28-day. UHPC shows a ductile behaviour for both tests due to the interaction between fibres and UHPC mix components. Table 4 shows the compressive strength and strain at peak stress for both cylindrical and cubical specimens.

Furthermore, the modulus of elasticity for UHPC was calculated based on two methods. The first method is based on scant modulus (*E*_0_), which is calculated based on the peak strength (maximum) and the corresponding strain. The second is based on the tangent modulus of elasticity (*E*_0_), which is calculated based on the stress and corresponding strain between 10% and 30% of the maximum compressive strength [3]. The cubical specimens show a higher modulus of elasticity and compressive strength when compared to the cylindrical specimen due to the shorter aspect ratio and larger lateral confinement provided by the machine plates, the same trend can be noticed in NSC [3]. Table 4 shows the results for both the secant and tangent modulus of elasticity for both cylindrical and cubical specimens.

The results from the experimental tests are used to calibrate the parameters that are needed for modelling UHPC. Table 5 lists the calibrated parameters for the fracture-plastic model. Figure 8 shows comparison between the experimental and numerical results. It should be noted that, for all numerical models, the mesh size of 13 mm (0.5 in.) was used. The mesh elements were hexahedra for cubical specimens, however, for cylindrical specimens, prism mesh element was used, except for the top and bottom portion, where quadrilateral mesh elements were required for geometrical requirements. The numerical models were run in displacement control steps of 0.127 mm (0.005 in.) with a total number of running steps equal to 100 steps. The Newton-Raphson method [62] was used as the solution method by setting the displacement error and residual error equal to 1% and number of iteration limit to 30. No convergence issues were observed for any model. All of the supports and loading plates were attached to the specimens with a fix-contact surface; in addition, the vertical and horizontal displacements of the supports were restricted.

### 5.2. Direct Tension and Flexural Tests

**Figure 9:**
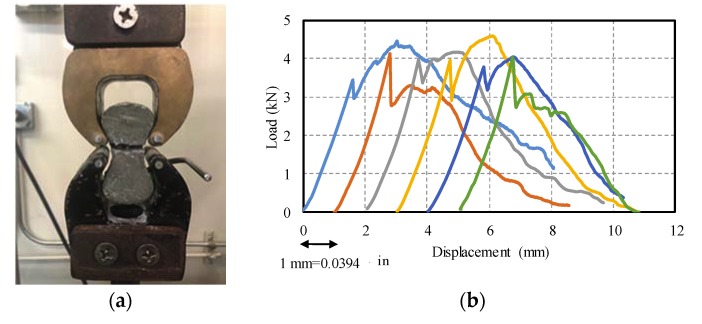
Direct tension test: (**a**) AASHTO T132 test; (**b**) load-displacement for briquettes. (1 kN = 0.22 kips).

The high compressive strength and high tensile strength [3,67,68], shorter reinforcement development length [69], and shorter lap splice length [70] are the main advantages of UHPC when compared to NSC. The tensile behaviour of UHPC before and after cracking was investigated under direct tension and flexural tests. For the direct tension test, six dog bone shape briquettes were tested according to AASHTO T132 [71]. The dimension of test specimens is 76.2 mm (3 in.) in length, 25.4 mm × 25.4 mm (1 in. × 1 in.) in cross-section at the middle, and 25.4 mm (1 in.) in thickness. The loading rate that was used in this test was 0.0254 mm/sec (0.001 in/sec.) according to [3]. Figure 9a shows one of the test specimens after testing. The testing of briquette specimens shows that UHPC behaved linear elastic before the first crack and then stress hardening occurred because the post-cracking resistance is higher than the resistance of the mixture. In this case, when the initial crack happened, the fibres would carry the tensile stress, which is known as the “bridge effect”. The average tensile cracking strength that was measured for UHPC in this series of tests was 3.8 kN (0.85 kips), with standard deviation and coefficient of variance of 0.2 kN (0.045 kips) and 0.06, respectively, with post cracking peak strength of 4.2 kN (0.94 kips) with a standard deviation and coefficient of variance of 0.2 kN (0.045 kips) and 0.05, respectively (Figure 9b). In Figure 9b, the results were only plotted with an offset of 1 mm (0.0394 in.) to better represent the results; however, the results, in reality, are not with an offset.

It should be noted that this test is not considered directly in the simulation because of the dimension of the briquette and effect of boundary condition, and it was just used as the preliminary data for simulating the flexural test.

**Figure 10:**
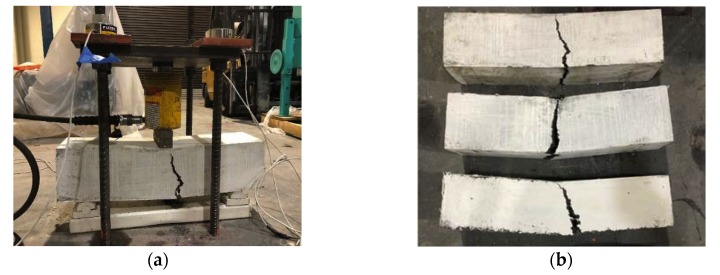

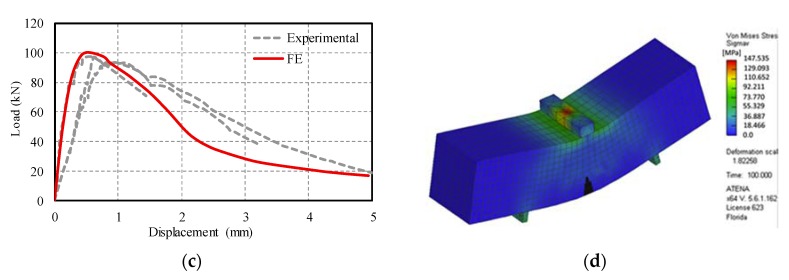
Flexural test: (**a**) test setup; (**b**) specimens after testing; (**c**) comparison between experimental and finite element results; and, (**d**) stress distribution in finite element model (1 kN = 0.22 kips, 1 in = 25.4 mm).

Although the uniaxial test can be considered to be the most realistic method for determining the tensile post-cracking behaviour, it has some difficulties, such as the boundary condition of the testing machine, the complicacy in the test setup and data collection [4], difficulties in obtaining evenly distributed stresses through the section, and controlling the stable load versus displacement/crack opening [5]. In this series of tests, the flexural test was conducted according to ASTM C1018 [72] on three small scale beams with a cross-section area of 153 mm × 153 mm (6 in. × 6 in.), a total length of 612 mm (24 in.), and an effective span length of 459 mm (18 in.). The small scale beams were supported over roller supports and they were tested using a three-point load test setup while using a hydraulic jack with a loading rate of 110 N/s (24.7 (lb/s)), as shown in Figure 10a. From the experimental results, the UHPC beam specimens show linear behaviour before the occurrence of the first crack and then the beam deformation was localized in the first crack with a nonlinear increase in deformation until failure, as shown in Figure 10b. The steel fibres could resist the tensile forces from the external load after the growth of the first crack that kept the beam specimen intact. The results from flexural tests were used to define the fracture-plastic model parameters of UHPC in tension, as shown in Table 5. A comparison between the experimental results and finite element results shows good correlation in force-displacement curves, as shown in Figure 10c. Figure 10d shows the numerical stress distribution showing the first crack propagation, which is comparable to the mode of failure in Figure 10b.

## 6. Results of Bond Strength Modelling

In the last step, the interface model was used to simulate the interfacial bond strength between UHPC and NSC. Table 6 shows the parameters that were related to the bond model for smooth and rough surfaces. These parameters are calculated by calibrating the experimental results of bi-surface shear tests and from literature. For this series of tests, the average surface roughness of sandblasted and smooth surfaces was measured as 1.2–2.2 mm (0.05-0.08 in.) and 0.17–0.28 mm (0.0067–0.110), respectively [39].

The bond tension strength (*f_t_*) was assumed from the direct pull-off test result from literature [37], bond cohesion (C) was calculated from a test with pure shear stresses and no normal stresses condition; however, in this model, the value, as calculated from the bi-surface shear test, was input directly into software as a reasonable approximation. The recommended value from AASHTO-LRFD was used for the coefficient of friction (μ) [73]. Normal stiffness *K_nn_* and tangential stiffness *K_tt_* are calibrated based on the experimental test results. Additionally, it should be noted that the minimum normal stiffness *K_nn(min)_* and minimum tangential stiffness *K_tt(min)_* that represent the interface stiffness after failure are chosen as 0.1% of the initial values to eliminate the numerical errors [63].

**Figure 11:**
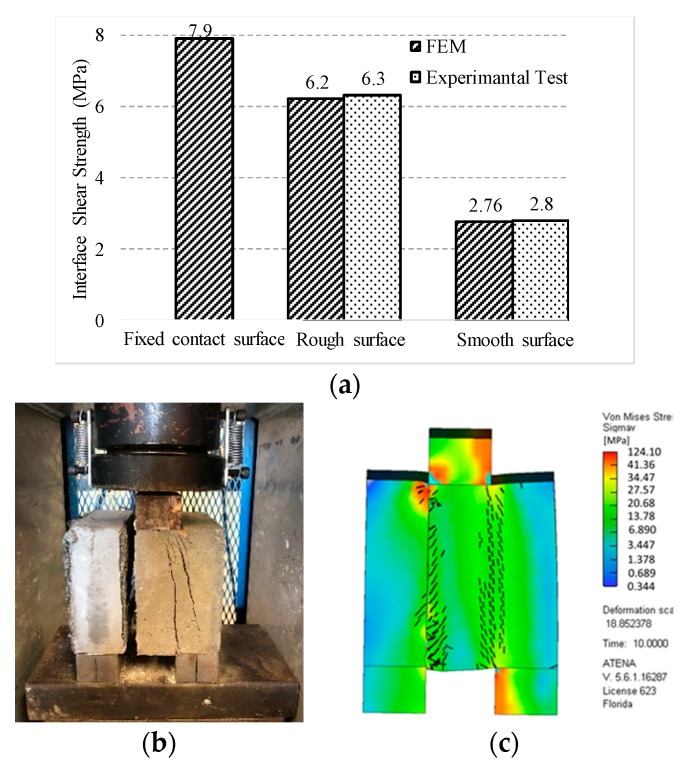
Bond strength numerical results: (**a**) Comparison between experimental and numerical model results of bond strength; (**b**) specimen failure; and, (**c**) stress distribution in the finite element model. (1MPa = 145 psi).

The results of the model with experimental and the model with a fixed contact for surface between the two layers of UHPC and NSC are compared in Figure 11a to highlight the importance of simulating the interface. Figure 11c shows the stress distribution from the finite element and its corresponding actual mode of failure from experimental testing (Figure 11b).

## 7. Summary and Conclusions

In this paper, the interfacial bond strength between normal strength concrete and ultra-high performance concrete with smooth and rough interface surfaces was experimentally and numerically investigated. First, the interfacial bond strength was evaluated experimentally by testing 10 cubical specimens using a bi-surface shear test setup. Second, 18 different test specimens, including cylinders, cubes, briquettes, and flexural beams, were cast and tested under compression, direct tension, and flexural tests to calibrate the UHPC material model in ATENA FE software. In the end, the results from both experimental and numerical results were used to calibrate the parameters of a zero thickness volume interface model in FE software. The numerical results were compared with the experimental results and the conventional fixed contact model approach. The following conclusions and observations can be drawn based on the conducted research:

- In ambient conditions and after 28-day, the compressive strength and tensile strength of UHPC reached 126 MPa (18 ksi) and 6.5 Mpa (0.95 kips), respectively, which nominates UHPC as an efficient repair material for damaged structures.
- The bi-surface shear test results showed an average bond strength of 2.9 MPa (420 psi) for specimens with smooth interface surfaces, whereas this value increased by 134% for specimens with rough interface surfaces by sandblasting with an average surface roughness between 1.2–2.2 mm (0.05–0.08 in.).
- The plastic-fracture model could predict the tensile and compressive behaviours of UHPC with acceptable accuracy, which makes it a practical tool for modelling structures, including UHPC.
- For modelling the interface between UHPC and normal strength concrete, the result from the bi-surface test could be directly used as the interface cohesion parameter; however, the only calibrated parameters were the normal stiffness *K_nn_* and tangential stiffness *K_tt_*.
- Modelling of the interface using a fixed contact for the surface model cannot distinguish the effect of surface preparation on bond strength between normal strength concrete and UHPC, which might lead to erroneous numerical results.
- By comparing the fixed contact model and the zero thickness volume model with experimental results, the error of simulation for smooth and rough surface dropped from 182% and 25% to around 1%, respectively.

## Figures and Tables

**Table 1:** UHPC mixture proportions. (1 kg = 2.2 lb) [3,39].

Constituent		Portion Based on Each Premix Bag (Kg)	Percentage by Weight (%)
Ductal^®^ JS1000	Portland cement	7.43	28.5
	Fine sand	10.64	40.8
	Ground quartz	2.2	8.4
	Silica fume	2.41	9.3
	Accelerator	0.31	1.2
	Total weight of premix	23	88.2
Water		1.2	4.4
Steel fiber 2%		1.6	6.2
Superplasticizer		0.32	1.2

**Table 2:** UHPC mixing procedure [3,39].

Procedure	Start Time (min)
Mixing UHPC dry premix	0
Adding water	4
Adding half superplasticizer	4
Adding the other half superplasticizer	19
Adding steel fibers	21
Mixing until complete uniformity	≃30

**Table 3:** Rheological property measurement based on ASTM C1437 [48]. (1 in. = 25.4 mm.).

Spread Diameter after 20 Drops (mm)	Mix Rheology
<200	Stiff
200–250	Fluid
>250	Highly Fluid

**Table 4:** Experimental stress-strain results for the test specimens under compressive test (1 MPa = 145 psi).

Specimen Type	Property	Average (MPa)	Standard Deviation (MPa)	Coefficient of Variance %
Cylinderical Specimen after 28 days	Compressive strength	126	3	7.3
	Strain at peak stress	0.00353	0.000510	0.007
	Secant elastic modulus, *E*_0_	36,016	3153.5	8.8
	Tangent elastic modulus, *E*	52,081	4136.9	7.9
Cubical Specimen after 28 days	Compressive strength	173	5.0	12.45
	Strain at peak stress	0.00408	0.000258	0.002
	Secant elastic modulus, *E*_0_	42,560	4095.0	9.6
	Tangent elastic modulus, *E*	61,191	3909.3	8.1

**Table 5:** Calibrated UHPC plastic-fracture model parameters based on experimental test results in ATENA software. (1 MPa = 145 psi, 1 in. = 25.4 mm).

Elastic Zone			
Modulus of elasticity	52,081 MPa	Poisson’s ratio	0.2
Compressive strength	−126 MPa	Tensile strength	5.8 MPa
**Plastic Zone**			
Compression characteristic size	1.27 mm	Tension characteristic size	1.27 mm
Compression localization onset	−0.001	Tension localization onset	0.002
**Compressive behavior**		**Tensile behavior**	
Yield strain	Compressive stress	Crack strain	Tensile stress
0	−126 MPa	0	1.1 MPa
−0.001	−126 MPa	0.002	6 MPa
−0.01	−38 MPa	0.1	1 MPa

**Table 6:** The parameters of the interface model for different surface preparation calibrated from bi-surface shear test. (1 MN/m^3^ = 3.68 lb/in^3^).

	*C* MPa	*f_t_* MPa	Friction Coefficient	*K_tt_* (MN/m^3^)	*K_nn_* (MN/m^3^)	*K_tt(min)_* (MN/m^3^)	*K_nn(min)_* (MN/m^3^)
Sand blasted surface	6.28	2	1	2.2 × 10^8^	2.2 × 10^8^	2.2 ×10^6^	2.2 × 10^6^
Smooth surface	2.8	0.5	0.5	10^6^	10^6^	10^4^	10^4^
